# Barriers to clinical remission in severe asthma

**DOI:** 10.1186/s12931-024-02812-3

**Published:** 2024-04-24

**Authors:** Inês Farinha, Liam G Heaney

**Affiliations:** 1 Pulmonology Department, Coimbra Hospital and University Centre, Praceta Prof. Mota Pinto, Coimbra, 3004-561 Portugal; 2 Wellcome-Wolfson Institute for Experimental Medicine, 97 Lisburn Road, Belfast, BT9 7BL Northern Ireland, UK

**Keywords:** Asthma, Clinical remission, Biologics

## Abstract

Severe asthma is associated with an increased risk for exacerbations, reduced lung function, fixed airflow obstruction, and substantial morbidity and mortality. The concept of remission in severe asthma as a new treatment goal has recently gained attention due to the growing use of monoclonal antibody therapies, which target specific pathologic pathways of inflammation. This review evaluates the current definitions of asthma remission and unveils some of the barriers for achieving this state in the severe asthma population. Although there is no unified definition, the concept of clinical remission in asthma should be based on a sustained period of symptom control, elimination of oral corticosteroid exposure and exacerbations, and stabilization of pulmonary function. The conjugation of these criteria seems a realistic treatment target in a minority of asthmatic patients. Some unmet needs in severe asthma may affect the achievement of clinical remission. Late intervention with targeted therapies in the severe asthma population may increase the risk of corticosteroid exposure and the development of irreversible structural airway changes. Moreover, airway infection is an important component in persistent exacerbations in patients on biologic therapies. Phenotyping exacerbations may be useful to guide therapy decisions and to avoid the liberal use of oral corticosteroids. Another challenge associated with the aim of clinical remission in severe asthma is the multifaceted interaction between the disease and its associated comorbidities. Behavioural factors should be evaluated in case of persistent symptoms despite optimised treatment, and assessing biomarkers and targeting treatable traits may allow for a more objective way of reaching remission. The concept of clinical remission will benefit from an international consensus to establish unifying criteria for its assessment, and it should be addressed in the future management guidelines.

## Background

Asthma is the most common noncommunicable respiratory disease, affecting an estimated 339 million people globally. It is a heterogeneous disease characterised by chronic airway inflammation, variable airflow limitation, mucus hypersecretion and bronchial smooth muscle hyperresponsiveness, with variable symptoms over time [1]. Severe asthma, comprising approximately 3–10% of all patients, is defined by the European Respiratory Society/American Thoracic Society task force as asthma that requires high-dose inhaled corticosteroids plus a second controller and/or oral corticosteroids (OCS) to remain controlled, or that remains uncontrolled despite this therapy [2–4]. It is associated with an increased risk for exacerbations, reduced lung function and fixed airflow limitation, daily symptoms, and adverse effects of treatment, especially those from OCS [4–9]. Severe asthma is associated with considerable morbidity and mortality, and a significant use of healthcare resources [1, 10]. Inhaled and oral corticosteroids have been the cornerstone of asthma therapy for many years, but they have not been associated with a significant long-term impact on the course of the disease [11]. Knowledge of the underlying pathophysiological changes in asthma has improved significantly during the last decades, allowing for the characterisation of disease phenotypes and the introduction of targeted biologic therapies [3, 12]. The current treatment goals are still focused on symptom control and reduction of future exacerbation risk [12].

The concept of disease remission is important in medicine and it may be described as a state of low to no disease activity for a defined period of time, and it can be spontaneous or a result of therapy [4, 11, 13]. To date, the concept of remission in asthma has mostly been described as the spontaneous disease activity cessation in childhood asthma, which is a common phenomenon [4, 11, 12, 14–16]. Although the term ‘remission’ is rarely used in the current management of adults with asthma, other chronic inflammatory diseases such as rheumatoid arthritis, ulcerative colitis and psoriasis have established definitions for disease remission, mainly associated with the introduction of disease-modifying drugs and targeted biologic therapies [4, 11, 12, 17–19]. Indeed, the management paradigm in these diseases has shifted from ‘treat-to-failure’, in which therapy is sequentially increased in a non-targeted way to the maximum recommended dose when clinical improvement is not reached, to ’treat-to-target’, whereby key pathologic pathways are identified and targeted, aiming to induce sustained disease remission or sustained reduction in disease activity [4, 11, 20]. The arrival and increasing use of monoclonal antibody therapies in the clinic for severe asthma has now raised the possibility of disease remission as a new treatment target and has recently gained attention. In this review, we assess the current concepts of asthma remission and some limitations for achieving remission in the severe asthma population [11, 12].

## Clinical remission in asthma

The definition of remission in asthma should be both comprehensive and practical and it should address multiple impacts of the disease across the whole spectrum of severity, in order to improve the existing concept of asthma control [1, 7, 12]. It should be based on daily asthma symptoms, exacerbation frequency, future exacerbation risk, pulmonary function and laboratory markers of inflammation, and it should require an adequate duration of assessment to address variability (including seasonality) of disease activity [1, 11].

### Proposed definitions of remission

Menzies-Gow et al. used a modified Delphi survey approach to propose a consensus framework for the key components for a definition of clinical remission in asthma [4, 11]. Evidence from other chronic inflammatory diseases (rheumatoid arthritis, Crohn’s disease, ulcerative colitis, and systemic lupus erythematosus), with recognized definitions of remission as a treatment target, was used as the basis for the consensus [11, 12]. The clinical experts identified four subtypes of asthma remission definitions (clinical and complete remission, both on and off treatment) [11, 12]. The clinical remission framework required at least 12 months of absence of significant asthma symptoms, optimization/stabilization of lung function, no OCS use for asthma, and healthcare professional/patient agreement that disease remission had been achieved [4, 11]. Upham et al. also used a Delphi process to develop a consensus for defining a ‘super-responder’ in severe asthma [16]. The super-response definition involved improvements in three or more criteria (at least two of which should be major criteria) assessed over 12 months. Major criteria included: exacerbation elimination, major improvement in asthma control (≥2x minimal clinically important difference improvement in asthma control using a validated instrument), or cessation of maintenance OCS or weaning to adrenal insufficiency. Minor criteria included: 75% exacerbation reduction, ≥500 mL improvement in forced expiratory volume in 1 s (FEV_1_), or achievement of well-controlled asthma (Asthma Control Questionnaire (ACQ) < 1.0 or Asthma Control Test (ACT) > 19) [16]. Several national societies have proposed other definitions of asthma control and remission as treatment targets. In the Japanese guidelines for adult asthma, the concept of ‘well-controlled’ disease requires the presence of no asthma symptoms, no use of reliever therapy, no limitation of activities (including exercise), FEV_1_ and peak expiratory flow (PEF) ≥ 80% of the predicted value or personal best value, < 20% of diurnal (weekly) variation of PEF and no exacerbations (including no unscheduled visits, emergency department visits or hospitalizations) [21]. The concept of ‘asthma remission’ in the latest German Respiratory Society guidelines requires a sustained (≥12 months) absence of asthma symptoms and exacerbations, stable lung function, and no need for OCS for the treatment of asthma [22]. The Severe Asthma Network Italy (SANI) definition of ‘complete clinical remission’ requires a period of ≥12 months of absence of OCS use and three additional criteria: absence of asthma symptoms (ACT score 20–25 and ACQ score < 1.5), absence of exacerbations, and stability of lung function. An alternative definition of ‘partial clinical remission’ is also contained in these guidelines when only two of the three last criteria are achieved [23]. Another definition of ‘clinical remission’ defined by the American College of Allergy, Asthma, and Immunology (ACAAI), American Academy of Allergy, Asthma, and Immunology (AAAAI), and American Thoracic Society (ATS) workgroup requires all of the following criteria over a period of 12 months: no exacerbations requiring a physician visit, emergency care, hospitalizations and/or systemic corticosteroid for asthma; no missed work or school over a 12-month period due to asthma-related symptoms; stable and optimized lung function results on all occasions, when measured over a 12-month period, with at least two measurements per year; continued use of controller therapies (ICS, ICS/LABA, leukotriene receptor antagonist) only at low-medium dose of ICS or less, as defined by most recent GINA strategy; an ACT > 20, AirQ < 2, ACQ < 0.75 on all occasions measured over the previous 12-month period, with at least two measurements per year; and symptoms requiring one-time reliever therapy (SABA, ICS-SABA, ICS-LABA) no more than once a month [24]. As this definition requires six mandatory criteria, which are often hard to achieve in clinical practice, it is by comparison the most ambitious interpretation of remission, and this will substantially impact the number of patients who can potentially reach this state.

### Evidence of remission from clinical trials

Some studies have analysed the proportion of patients who meet these criteria. A *post-hoc* analysis used the results from three previous phase 3 trials of benralizumab (SIROCCO, CALIMA, and ZONDA) with the aim of identifying patients who achieved some or all the criteria for clinical remission in severe asthma: zero exacerbations, zero OCS use, asthma control (ACQ-6 score < 1.5 OR ≤ 0.75), and improvement in lung function (pre-bronchodilator (BD) FEV_1_ increase ≥100 mL) [4]. Across all three trials, about three quarters of the patients (87% in the SIROCCO/CALIMA trials and 75% in the ZONDA trial) achieved two or more components of clinical remission and approximately half achieved three or more remission components at the 6-month timepoint since beginning treatment with benralizumab. The response rates were similar after 12 months of treatment. Overall, 15 to 23% of patients achieved clinical remission within 6 months, and approximately 15% achieved remission after 12 months of therapy. Notably, a substantial proportion of patients in the placebo arm also achieved clinical remission, suggesting that taking part in a clinical trial with regular review, probably better treatment adherence and the ‘placebo effect’ can also deliver clinical remission [4]. In a similar *post-hoc* analysis with dupilumab, 20.1% of patients with moderate to severe asthma achieved clinical remission (defined as OCS free, ACQ < 1.5 and FEV_1_(%) > 80% predicted) but again a smaller proportion (4.6%) achieved remission in the placebo arm [25]. In the NAVIGATOR study, tezepelumab demonstrated 12.7% of patients with moderate to severe asthma achieved clinical remission compared to 4.4% with placebo [26].

Biologic therapies can achieve greater clinical remission responses compared to placebo but only in a minority of patients with moderate to severe asthma. It is also worth commenting that the definition of clinical remission was different in each of these analyses, particularly on the issue of lung function, and it will be important going forward to have a unified agreed definition to allow comparisons across clinical trials.

### Evidence of remission from real life studies

Clinical trial populations are very different from real-world populations and one UK study demonstrated that 9.8% (range 3.5–17.5%) of UK severe asthma patients would have been eligible for enrolment in the phase 3 trials of biologic therapies in severe asthma [27]. Thus, it is important to understand clinical remission outcomes in real-world populations. The REal worlD Effectiveness and Safety (REDES) study was a real-life study performed in Spain that aimed to evaluate the effectiveness and safety of mepolizumab in severe eosinophilic asthma and included a patient stratification by baseline blood eosinophil count [3]. The primary endpoint was the change in the annual rate of clinically significant asthma exacerbations, defined as those requiring the administration of a systemic corticosteroid for at least 3 days (or doubling the dose in patients on maintenance OCS), or if the patient had visited an emergency department or was hospitalized. A total of 318 patients were included, with a predominance of women (*n* = 220, 69.2%) and a mean (SD) age of 56.5 (12.5) years. The rate of exacerbations was significantly reduced by 77.5% during the year post-mepolizumab introduction (*p* < 0.001) – from a mean (SD) of 4.5 (3.5) exacerbations per year before mepolizumab, to 1.0 (1.4) per year after beginning mepolizumab. All baseline eosinophil subgroups reduced the mean number of exacerbations, irrespective of the eosinophil count. This study revealed that mepolizumab was safe and effective in improving asthma control and reducing severe exacerbations and need for OCS. A subsequent analysis which examined clinical remission in a subset of 144 patients with complete data (OCS use, ACT score, and FEV_1_ ≥80% at 1-year post-mepolizumab initiation) revealed that approximately 30% achieved all these criteria [3]. Hansen et al. recently assessed the rate of ‘clinical remission on biologic treatment’ – defined as absence of exacerbations and maintenance OCS, normalization of lung function (FEV1% >80%) and an ACQ score ≤ 1.50 – based on data from the Danish Severe Asthma Registry and found that 19% (43 out of 225 of clinical responders) met these criteria [28]. In another study which included data from two real-world drug registries – the Australian Mepolizumab Registry (AMR) and the Australian Xolair Registry (AXR) –, clinical remission was found in 29.3% (73/249) and 22.8% (37/162) of cohorts, respectively. The definition included no exacerbations and no OCS use during the previous 6 months assessed at 12 months and ACQ-5 ≤ 1 at 12 months. When lung function criteria were added at the definition – optimization (post-bronchodilator FEV_1_ ≥ 80%) or stabilization (decline in post-bronchodilator FEV_1_ ≤ 5% from baseline) at 12 months –, the proportion of clinical remission reduced to 25.2% and 19.1%, respectively [29]. The UK Severe Asthma Registry (UKSAR) is the largest national database of severe asthma and contains demographic, clinical, and treatment characteristics of patients with uncontrolled asthma referred to specialist UK severe asthma centres [30]. Biologic access for severe asthma in the UK is restricted on the basis of cost-effectiveness by the National Institute of Clinical Healthcare Excellence (NICE), so that only patients on maintenance corticosteroids for disease control or requiring 3 or more rescue courses of prednisolone per year can be eligible for a biologic. This allows UKSAR to assess the outcome of clinical remission within a cohort of well-characterised patients with severe asthma with substantial OCS exposure. A recent study by McDowell et al. aimed to evaluate the endpoint of clinical remission, primarily defined as ACQ < 1.5 and no OCS for disease control (no OCS bursts, no maintenance OCS for disease control (except OCS ≤ 5 mg/day for hypothalamic-pituitary-adrenal (HPA) axis suppression), and an FEV_1_ ≥ lower limit of normal (LLN) or no more than 100mL less than pre-biologic FEV_1_), in a cohort of patients with severe asthma from the UKSAR database [31]. Sensitivity analysis was also applied to assess the impact of different definitions of clinical remission, including the addition of SABA use and different FEV_1_ parameters (including the exclusion of FEV_1_). A total of 1111 patients from 14 severe asthma centres in the UK were included. Among the study population, 18% of patients met the primary definition of clinical remission one year after the beginning of biologic therapy. Remission was associated with the following characteristics: male sex, older age (55.0 (48.0,65.0) vs. 51 0.0 (41.0,59.0) years, *p* < 0.001), shorter duration of symptoms (20.0 (8.0,32.0) vs. 24.5 (12.0,37.0) years, *p* = 0.008), never smoking, nasal polyps, white ethnicity, lower body mass index (BMI) (27.9 (25.4,31.8) vs. 30.5 (26.6,35.1)kg/m^2^, *p* = 0.001), and T2 composite high (blood eosinophil count (BEC)/FeNO) prior to beginning biologics (FeNO (51.0 (35.0,81.0) vs. 41.0 (22.0,72.0) ppb, *p* = 0.002) and highest-recorded BEC (0.79 (0.58,1.33)x10^9 vs. 0.68 (0.40,1.00), *p* < 0.001)). Non-remission was associated with a higher incidence of depression-anxiety (12.5% vs. 2.0%, *p* < 0.001), higher number of exacerbations (5 (3.8) vs. 4 (3.6), *p* < 0.001), emergency department attendances (39.2% vs. 23.3%, *p* < 0.001) and hospital admissions in the previous year (40.5% vs. 30.4%, *p* = 0.022), higher baseline symptom burden (ACQ-5 3.2 (2.2,4.2) vs. 2.0 (1.2,3.4), *p* < 0.001) and more impaired quality of life. Regardless of the definition applied for clinical remission, only a minority of patients (12–21%) met the criteria.

In summary, the concept of clinical remission in severe asthma seems an achievable treatment goal only in a minority of patients. Symptom control, weaning of OCS use, reduction in exacerbations are important criteria to be included in the definition of clinical remission. Understanding the different characteristics seen in remission vs. non-remission groups may allow for a better clarification and standardization of the concept.

## Barriers to achieving remission

### Late intervention with effective treatment

One of the most important benefits of biologics is their success in reducing or eliminating OCS exposure. There is an increased incidence of corticosteroid toxicity in individuals with severe asthma exposed to glucocorticoids and recent data has demonstrated that at population level, corticosteroid induced morbidity is seen at low cumulative exposure levels. A UK study demonstrated most morbidities increased an average of one prescription per year, a cumulative dose < 500 mg (two short courses) and/or an average daily dose of ≤ 1 mg in prior two years [32] and a US study confirmed multiple morbidities with increasing cumulative burden of OCS exposure [33]. Thus, given the association of OCS morbidities such as anxiety / depression and obesity with a reduction in rates of remission, earlier intervention to try and prevent these OCS related comorbidities will be important to improve outcomes [33–37]. A post-hoc analysis of the REDES study revealed that patients who achieve clinical remission tend to have a lower burden of symptoms (mean ACT score 15.2 vs. 13.6), better lung function (mean post-BD FEV_1_ 82.2% vs. 73.6%), lower use of long-term OCS (21% vs. 52%), and lower dose of maintenance OCS (median OCS dose 5.0 mg/day vs. 10.0 mg/day). These results suggest that patients with less severe disease may have a greater chance of achieving remission and prolonged corticosteroid exposure may be a significant barrier to clinical remission [3]. McDowell et al. performed a real-world prospective cohort study of 101 severe eosinophilic asthma patients treated with mepolizumab over a 12-month period to quantify change in glucocorticoid-associated toxicity using a validated tool (Glucocorticoid Toxicity Index – GTI) [35]. Although they found a significant reduction in OCS, the degree of toxicity change varied widely, with 27% experiencing an increase in toxicity and 40% of patients failing to meet the minimal clinically important difference for the GTI [35]. In a 3-year follow-up of this cohort, those patients who failed to show toxicity reduction at 1 year continued to have progressive worsening toxicity at year 3 despite effective OCS reduction, suggesting the opportunity to alter trajectory in terms of OCS toxicity had been missed [35]. These studies reinforce the need for early targeted intervention to prevent prolonged and often inappropriate exposure to OCS and progressive corticosteroid-induced toxicity.

### Persistent exacerbations on biologic therapy

Biologic therapies have shown to be effective in reducing exacerbation rate in severe asthma [10]. The MEX study aimed to evaluate the inflammatory profile of residual exacerbations from a cohort of patients with severe eosinophilic asthma who were treated with mepolizumab. The authors found that these exacerbations are heterogeneous in nature, with approximately half driven by eosinophilic inflammation (despite very significant suppression of blood eosinophil count) and half being non-eosinophilic events [38]. These two exacerbation phenotypes were mutually exclusive and fractional exhaled nitric oxide (FeNO) at exacerbation was found to be a useful and straightforward tool to discriminate between high and low sputum eosinophils in patients treated with mepolizumab. Authors suggest that careful consideration should be given before administration of OCS when FeNO is low (≤ 20 ppb), given the NPV of 100% (95% CI 0.8-1.0) for an eosinophilic event. On the other hand, OCS are likely to be indicated when FeNO is high (≥ 50 ppb), with a PPV 76.9% (95% CI 0.6–0.9) [38]. Neutrophilic exacerbations are more likely to be pathogen-driven, with the evidence of virus or bacteria (*Haemophilus influenzae, Moraxella catarrhalis, Streptococcus pneumoniae, and Staphylococcus aureus*) [39].

A prospective study by Poznanski et al. was performed to evaluate whether a sub-optimal response to benralizumab, defined as either the maintenance of exacerbations or failure to reduce prednisone by at least 50%, might be associated with inadequate suppression of blood and sputum eosinophilia, due to impaired natural killer (NK) cell number/function [40]. A total of 74 severe asthma patients treated with benralizumab were included, 23 of them had circulating NK cells enumerated by flow cytometry. Sub-optimal response to benralizumab was obtained in 27% of patients during a median treatment period of 14 months. Most exacerbations in this subgroup of patients were neutrophilic (mean sputum neutrophils 10^6^/gm ± SD: responder = 4.5 ± 6 vs. sub-optimal responder = 19.5 ± 37, *p* = 0.01) and associated with infections. The number of infections in the preceding year in the sub-optimal response group (median 0.5, max-6; min-0) was significantly greater than in the response group (median 0, max − 2; min-0). A smaller number of circulating NK cells was evident in the sub-optimal responders, but a significant increase in circulating NKT cells, known for their role in recruiting and activating neutrophils, was found in this subset of patients.

These studies suggest that there is heterogeneity in persistent exacerbations in patients on biologic therapy targeting T2-inflamamtory mechanisms. Phenotyping exacerbations has been viewed as a crucial step towards targeted and effective treatment and may contribute to remission in severe asthma [41].

### Barriers to achieving symptom control

**Fig. 1:**
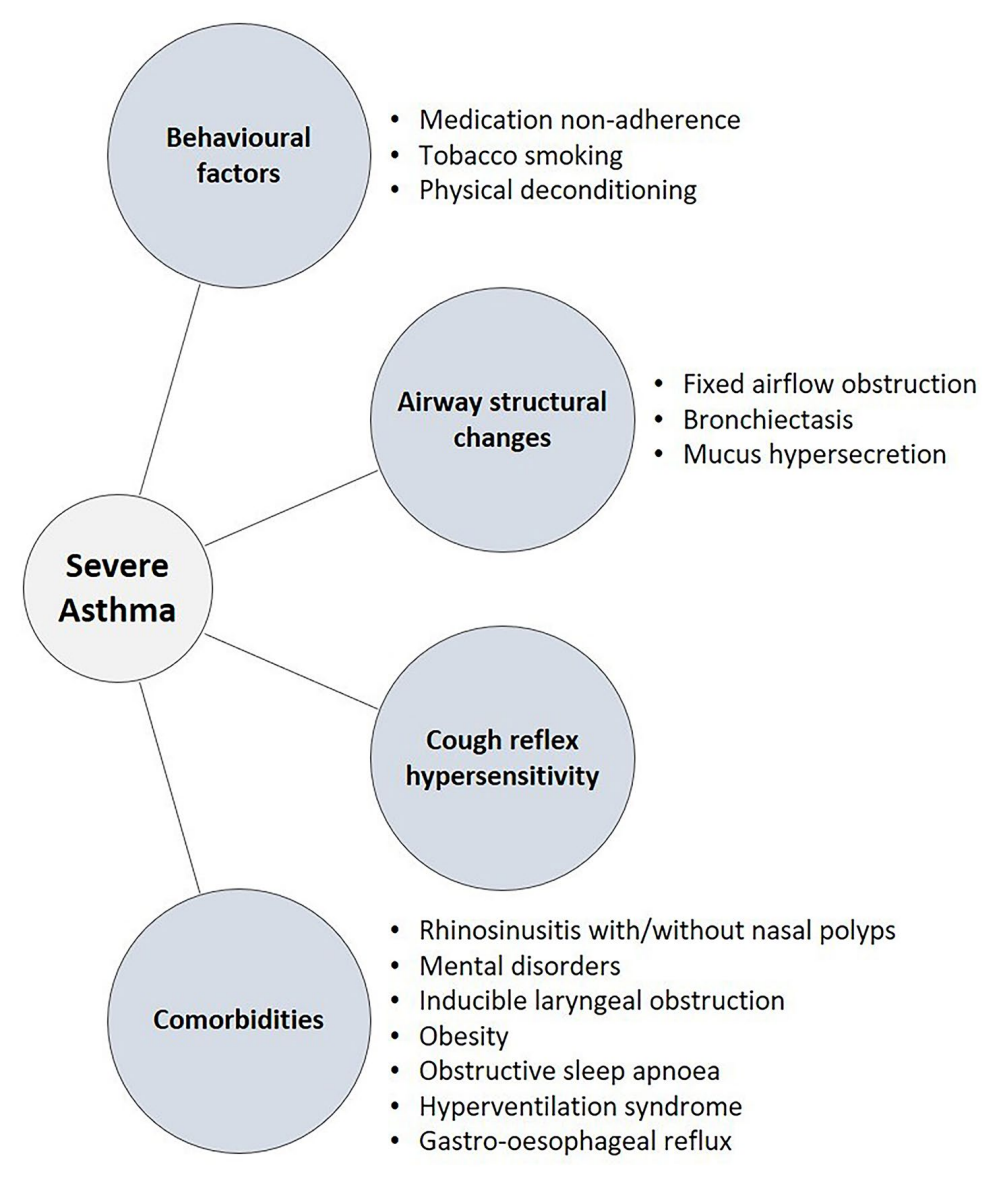
Factors associated with suboptimal symptom control in severe asthma

There are many factors associated with suboptimal symptom control in the severe asthma population, some of which are non-modifiable (Fig. 1) [12].

Some of the behavioural factors include medication non-adherence, tobacco smoking and obesity with physical deconditioning [42]. Achieving adherence is very important to prevent exacerbations. Non-adherence to ICS is one of the main issues to assess when considering ‘difficult-to-control asthma’, and persistently high FeNO has been associated with increased exacerbations in these patients [43, 44]. There has been a growing use of digital interventions such as mobile phones, text messages, and ‘smart’ inhalers which may help improve adherence and consequently asthma control and quality of life and reduce asthma exacerbations [42].

Airway structural changes, such as fixed airway obstruction, the presence of bronchiectasis and mucus hypersecretion may also challenge the achievement of remission in severe asthma. Different inflammatory pathways related to asthma require precision medicine approaches like ‘treat-to-target’ to increase the likelihood of success in reaching remission [4]. Cough reflex hypersensitivity has also been suggested as a cause of persistent symptoms and the imminent arrival of P2 × 3 antagonists in the clinic may make this a ’treatable trait’ in asthma [45]. A recent study investigated cough in patients with severe asthma stratified by composite T2-inflammatory biomarkers. The results showed that in patients with a composite T2-biomarker low profile (FeNO < 20 ppb and peripheral blood eosinophil count < 150 cells/µL) there appears to be a normal cough frequency and absence of cough morbidity, suggesting that suppression of T2-inflammation seems a logical initial step in treating cough in this population [46].

Asthma-related comorbidities are also important factors to take into consideration when it comes to the goal of achieving remission [47]. Conditions such as rhinosinusitis, nasal polyps, mood disorders, inducible vocal cord dysfunction, obesity, obstructive sleep apnoea, hyperventilation syndrome and gastro-oesophageal reflux need to be assessed and targeted in all patients.

There is a higher proportion of asthma in adult women (∼ 65% prevalence) and similarly there is a greater preponderance of females in severe asthma cohorts with high symptom burdens [48–50]. The UK Refractory Asthma Stratification Programme (RASP-UK) biomarker study evaluated symptom-based and biomarker-based corticosteroid adjustment in patients with severe asthma [43]. The patients with the higher symptom burden were predominantly female, had a higher BMI, were T2-biomarker low, were more likely to be on OCS, and were more likely to have corticosteroid-induced morbidities (gastro-oesophageal reflux, depression, and osteoporosis). A recent post-hoc analysis aimed to evaluate T2-composite-biomarker strategy to adjust corticosteroid treatment stratified by sex. A female preponderance was seen in both the overall study population (64.5% vs. 35.5%) and in uncontrolled asthma (ACQ-7 score ≥1.5: 70.7% female, 29.3% male) [47]. This study found that females derive greater benefit from biomarker-directed strategy to corticosteroid adjustment. This may be due to their higher symptom burden, which is associated with comorbid conditions such as obesity and mood disorders. In fact, targeting symptom control in this population of female severe asthmatic patients could expose them to excessive OCS use and their associated toxicities. Interestingly, when looking at factors mediating the sex difference in symptom burden, obesity, and anxiety and/or depression accounted for this difference [47].

The multifaceted nature of asthma and the complex interaction between this disease and its associated comorbidities driving persistent symptoms represents a challenge when it comes to aiming for a low symptom burden as currently defined by clinical remission. Measuring inflammation, targeting extra-pulmonary comorbidities – ideally by multidisciplinary teams –, and adopting a ‘treatable traits approach’ may be a more objective method and increase the proportion of patients achieving clinical remission [51, 52].

## Conclusion

Clinical remission in severe asthma may be an achievable goal in a minority of patients. The concept of clinical remission needs further discussion, and an international consensus will be important to establish unifying criteria for its assessment. Further studies are required on the efficacy of precision therapeutic approaches to evaluate the proportion of patients meeting the criteria for remission with these treatments. Future asthma guidelines should also focus on remission as a treatment goal.

Unmet needs in severe asthma should be addressed since they constitute barriers to clinical remission. Early intervention is crucial to reduce the onset of significant corticosteroid toxicity, and to delay disease progression and the development of structural changes. Airway infection is believed to be a significant player in persistent exacerbations on biologic therapy and other mechanisms may also be involved. Profiling exacerbations has been shown useful in tailoring therapy, thus questioning the liberal use of OCS. Behavioural factors and comorbid conditions should be evaluated in case of persistent symptoms despite optimised treatment.

## Data Availability

No datasets were generated or analysed during the current study.
